# Video‐Assisted Versus Conventional Surgical Informed Consent in Visceral Surgery—A Cluster Quasi‐Randomized Clinical Trial

**DOI:** 10.1002/wjs.70561

**Published:** 2026-09-05

**Authors:** Svenja G. P. D. Leicht, Leon Schuetze, Johan F. Lock, Christoph‐Thomas Germer, Johanna C. Wagner

**Affiliations:** ^1^ Department of General, Visceral, Transplantation, Vascular and Pediatric Surgery Medical Center Julius‐Maximilians‐University of Wuerzburg Wuerzburg Germany

**Keywords:** elective surgery, patient satisfaction, patient understanding, time efficiency, video‐assisted patient education

## Abstract

**Background:**

Comprehensive informed consent is a core surgical responsibility but is increasingly challenged by rising patient volumes and limited personnel resources. Digital solutions in healthcare offer opportunities to optimize patient education. Video‐assisted informed consent may reduce time expenditure without compromising informational quality. The objective was to compare video‐assisted with conventional informed consent regarding duration, patient understanding, patient, and physician satisfaction.

**Methods:**

In this prospective, cluster quasi‐randomized clinical trial at the University Hospital Wuerzburg, Germany (September 2023−March 2025), 153 of 330 screened patients scheduled for elective cholecystectomy or colon resection were enrolled. The intervention group received a video developed by *medudoc education GmbH*, while the control group received physician‐led informed consent according to the hospital's standard. Consultation duration was recorded using a stopwatch. Patients completed questionnaires on satisfaction and understanding immediately after consent and postoperatively, physicians rated satisfaction at study completion. The primary outcome was time reduction for the physician consultation time. Secondary outcomes included patient understanding, patient satisfaction, physician satisfaction, and postoperative pain.

**Results:**

Of 153 participants, 84 (55%) received video‐assisted and 69 (45%) conventional consent. Median physician consultation time was significantly reduced with video assistance for cholecystectomy (1.5 vs. 8.1 min) and colon resection (3.3 vs. 11.9 min). Patient understanding, satisfaction, and postoperative pain outcomes were high in both groups. Physician satisfaction with the video‐assisted process was consistently high.

**Conclusions:**

Video‐assisted surgical informed consent serves as a valuable adjunct in preoperative patient education and represents a promising approach to digital innovation in clinical practice.

**Trial Registration:**

ISRCTN registry ISRCTN26680260 (https://www.isrctn.com/search?q=ISRCTN26680260)

## Graphical abstract

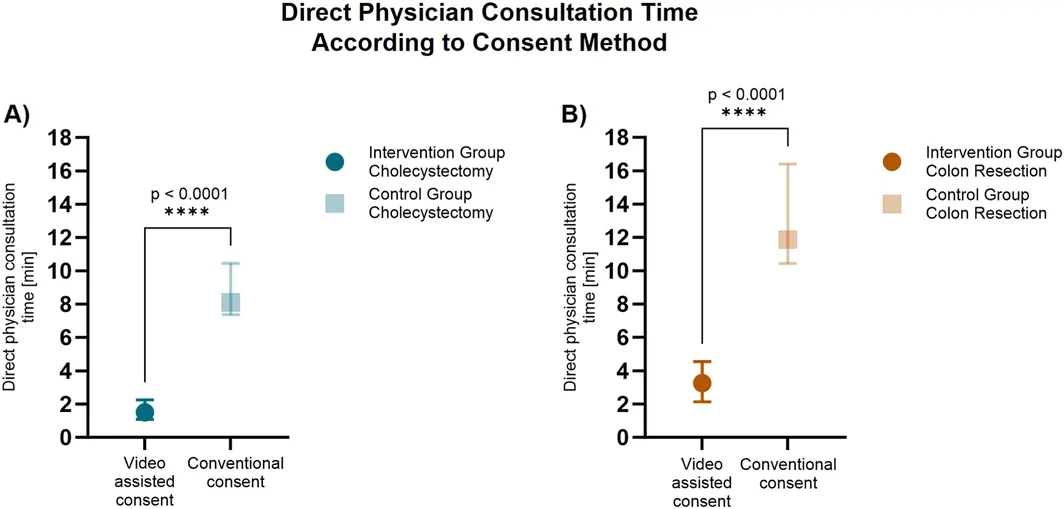

## 1 Introduction

Comprehensive surgical informed consent is one of the most fundamental responsibilities of a surgeon [1]. Preoperative informed consent, particularly in the context of elective procedures, is a prerequisite for performing invasive interventions and should provide comprehensive information regarding the surgical procedure, the medical necessity of the intervention, associated risks, and available alternatives [2, 3, 4, 5]. Conventionally, surgical informed consent is provided through a physician‐led consultation. However, due to many factors, such as fluctuating medical personnel, varying levels of clinical training, and differing professional and personal circumstances, this process remains highly non‐standardized [6]. As a result, the quality of information conveyed to patients can vary considerably, leading to differences in both patient understanding of the surgical procedure and satisfaction with the informed consent process [7, 8].

Thorough preoperative informed consent is time‐intensive and requires substantial physician resources, a resource that is becoming increasingly limited in the context of rising patient and surgical procedure numbers as well as a concurrent shortage of medical staff [9, 10]. In light of the growing availability of digital technologies in healthcare, multimedia‐based tools offer new opportunities to support and potentially standardize the surgical informed consent across different physicians and institutions [11, 12].

Video‐assisted informed consent has already been investigated in various surgical fields, including ophthalmology, otolaryngology, trauma surgery, dermatology, gynecology, and urology [3, 12, 13, 14, 15, 16, 17, 18, 19]. Clinical studies have demonstrated improvements in both patient comprehension and satisfaction with the preoperative discussion [3, 12, 13, 14, 15, 16, 17, 20, 21, 22, 23, 24, 25]. However, findings regarding the duration of informed consent when using video‐assisted formats are heterogeneous—ranging from comparable consultation times to significantly reduced durations [12, 15, 22, 24]. Previous trials comparing video‐assisted and conventional informed consent evaluated duration only exploratively. This trial primarily investigates the time required for different consent modalities.

Particularly for more complex surgical procedures, there is a pronounced need for thorough, standardized, yet individually tailored surgical informed consent. To date, video‐assisted surgical informed consent has not been systematically applied to abdominal surgery.

In light of the increasing number of patients and surgical procedures, and the resulting rise in demand for surgical informed consent paired with a concurrent shortage of medical personnel, this study primarily focuses on comparing the time requirements of conventional physician‐led versus video‐assisted surgical informed consent for visceral surgical procedures [9, 10]. Patient satisfaction with the surgical informed consent and patient understanding of the surgical intervention were assessed following both conventional and video‐assisted approaches. Additionally, physician satisfaction with the video‐assisted format was evaluated, an aspect that has not been a central focus in previously published clinical studies.

## 2 Methods

### 2.1 Design

This study was conducted as a prospective, cluster quasi‐randomized, single‐center clinical trial at University Hospital Wuerzburg, Germany, between September 2023 and March 2025. We hypothesized that video‐assisted surgical informed consent would reduce the duration of physician involvement in the informed consent process compared with conventional physician‐led informed consent. Patient understanding and satisfaction were assessed as secondary outcomes.

The unit of allocation was the study period, with all patients enrolled during the same study period receiving the same consent procedure. Consecutive study periods were allocated in an alternating manner to either conventional physician‐led informed consent (control group) or video‐assisted surgical informed consent (intervention group), with an intended allocation ratio of 1:1. The predefined allocation schedule was established and implemented by participating investigators who were also involved in patient recruitment. No random sequence generation or formal allocation‐concealment procedure was used. Because of the nature of the intervention, participating patients and physicians could not be blinded to group allocation. Outcome assessment and statistical analysis were not performed in a blinded manner.

### 2.2 Patients

Patients scheduled for elective cholecystectomy or colon resection were screened for eligibility. Study inclusion occurred after the indication for surgery had been confirmed during the physician consultation. Inclusion criteria were an age of at least 18 years, proficiency in the German language, and scheduled elective surgery. The only exclusion criterion was the occurrence of postoperative complications classified as Clavien‐Dindo grade IV or V.

### 2.3 Trial Procedures

Screening and study inclusion took place before elective cholecystectomy or colon resection, irrespective of the surgical indication or operative technique used, including robotic, laparoscopic, and open approaches. Colon resections included right and left hemicolectomy, transverse colon resection, and sigmoid resection.

Following inclusion, patients received either conventional physician‐led surgical informed consent in the control group or video‐assisted surgical informed consent in the intervention group using videos developed by *medudoc education GmbH*. For each patient in the intervention group, an individualized video was generated from modular sequences preselected by the attending physician according to the surgical indication and planned procedure. Patients could view the video as often as desired using hospital‐provided tablets and headphones.

After viewing the video, patients in the intervention group underwent a supplementary consultation with the physician to address any remaining questions and to obtain written informed consent for surgery. In the control group, patients underwent conventional physician‐led informed consent. After signing, they received a copy of the written consent form to take home. The duration of the conventional informed consent consultation in the control group and the supplementary physician consultation following video presentation in the intervention group was measured using a stopwatch. The content and structure of the physician‐patient consultations, including the number and type of patient questions, were not systematically recorded.

Following the informed consent process, patients completed questionnaires assessing their preoperative understanding of and satisfaction with the informed consent process. Postoperative questionnaires were administered to assess postoperative understanding and satisfaction during hospitalization. Patients, who developed severe postoperative complications classified as Clavien‐Dindo grade IV or V, were not asked to complete the postoperative questionnaires to avoid imposing an additional burden during critical illness. These patients remained eligible for analyses of the primary outcome and all other outcomes for which data were available.

In addition, postoperative pain was assessed during hospitalization using the Numeric Rating Scale (NRS).

After completion of the study, participating physicians received a questionnaire assessing their satisfaction with the video‐assisted surgical informed consent process, the number of video‐assisted informed consent procedures conducted, and the time required to generate each patient‐specific video.

### 2.4 Outcome Measures

The primary outcome was the duration of physician involvement in the informed consent process, measured using a stopwatch. This comprised the duration of the conventional informed consent consultation in the control group and the duration of the supplementary physician consultation following video presentation in the intervention group.

Secondary outcomes included preoperative and postoperative patient understanding and satisfaction, assessed using patient questionnaires, and physician satisfaction, assessed using a physician questionnaire. Questionnaire items were rated on a 4‐point Likert scale. Postoperative pain during hospitalization, assessed using the NRS, was an additional secondary outcome.

### 2.5 Statistical Analysis

The sample size calculation was based on the primary outcome of physician consultation duration. Assuming a medium standardized effect size (Cohen's *d* = 0.5) and a statistical power of 80%, an independent‐samples *t*‐test indicated that 51 patients per study arm were required. Accordingly, 102 patients were required for each surgical cohort. As the study included two surgical cohorts, cholecystectomy and colon resection, with separate comparisons between the intervention and control groups, the planned total sample size was 204 patients. The sample size calculation was performed at the individual‐patient level and did not account for the allocation of patients at the study‐period level. Accordingly, no adjustment based on an intracluster correlation coefficient or average cluster size was applied.

Statistical analyses were performed using GraphPad Prism, version 10 (GraphPad Software Inc., La Jolla, CA, USA). Between‐group comparisons were performed using unpaired t‐tests for normally distributed continuous variables and the Mann–Whitney *U* test for non‐normally distributed or ordinal variables. Categorical variables were compared using chi‐square tests or Fisher's exact tests, as appropriate. No statistical adjustment for clustering at the study‐period level was performed.

Data for consultation duration, age, body mass index, NRS scores, and questionnaire responses are presented as median with 95% confidence interval, unless otherwise stated. Effect estimates are reported with corresponding 95% confidence intervals. All tests were two‐sided, and *p* values below 0.05 were considered statistically significant.

All patients with an available measurement of physician consultation duration were included in the analysis of the primary outcome. Patients with Clavien‐Dindo grade IV or V complications were excluded only from analyses requiring postoperative questionnaire data if questionnaire completion was considered clinically inappropriate. No imputation of missing postoperative questionnaire data was performed.

### 2.6 Data Access Statement and Ethics Statement

The data supporting the findings of this study are available from the corresponding author upon reasonable request. Due to patient privacy regulations and restrictions imposed by the institutional review board, the datasets cannot be made publicly available.

This study was approved by the Ethics Committee of the Julius‐Maximilians‐University of Wuerzburg, Germany (Approval No. 112/22‐sc). All study procedures were conducted in accordance with the Declaration of Helsinki. Written informed consent was obtained from all participants.

## 3 Results

A total of 330 patients were screened, of whom 153 were enrolled in the study. Among the included patients, 107 underwent elective cholecystectomy and 46 underwent colon resection. Of the 107 patients scheduled for cholecystectomy, 59 (55%) received video‐assisted surgical informed consent and 48 (45%) received conventional physician‐led informed consent. Of the 46 patients scheduled for colon resection, 25 (54%) received video‐assisted informed consent and 21 (46%) received conventional physician‐led informed consent.

### 3.1 Patient Characteristics

Overall, 97 patients (63%) were female and 56 (37%) were male. The median age of the included patients was 58 years [55–62], and the median body mass index (BMI) was 27.3 kg/m^2^ [25.4–29.0]. No statistically significant differences in baseline patient characteristics were detected between the study groups (Table 1).

**TABLE 1:** Characteristics of the study population.

Group	Intervention (*n* = 84)		Control (*n* = 69)	
Operation	Cholecystectomy	Colon resection	Cholecystectomy	Colon resection
No. of patients (%)	59 (39)	25 (16)	48 (31)	21 (14)
Female (%)	42 (27)	13 (9)	28 (18)	14 (9)
Male (%)	17 (11)	12 (8)	20 (13)	7 (5)
BMI, median [CI], [kg/m^2^]	29.0 [26.3–30.9]	29.4 [24.9–32.9]	25.4 [24.2–27.4]	24.3 [22.7–28.1]
Age, median [CI], [years]	58 [55–63]	67.5 [59–72]	50 [41–56]	70 [55–74]

Abbreviation: BMI, body mass index.

### 3.2 Video‐Assisted Informed Consent Was Associated With Shorter Physician Consultation Duration

The primary finding was a substantial reduction in physician consultation duration among patients receiving video‐assisted informed consent. For Patients planes for a cholecystectomy, the median duration of the supplementary physician consultation following video presentation was 1.5 min [1.1–2.3], compared to 8.1 min [7.4–10.5] for conventional physician‐led informed consent. This corresponded to a difference of 6.6 min between the group medians (****p < 0.0001; Figure 1A).

**FIGURE 1:**
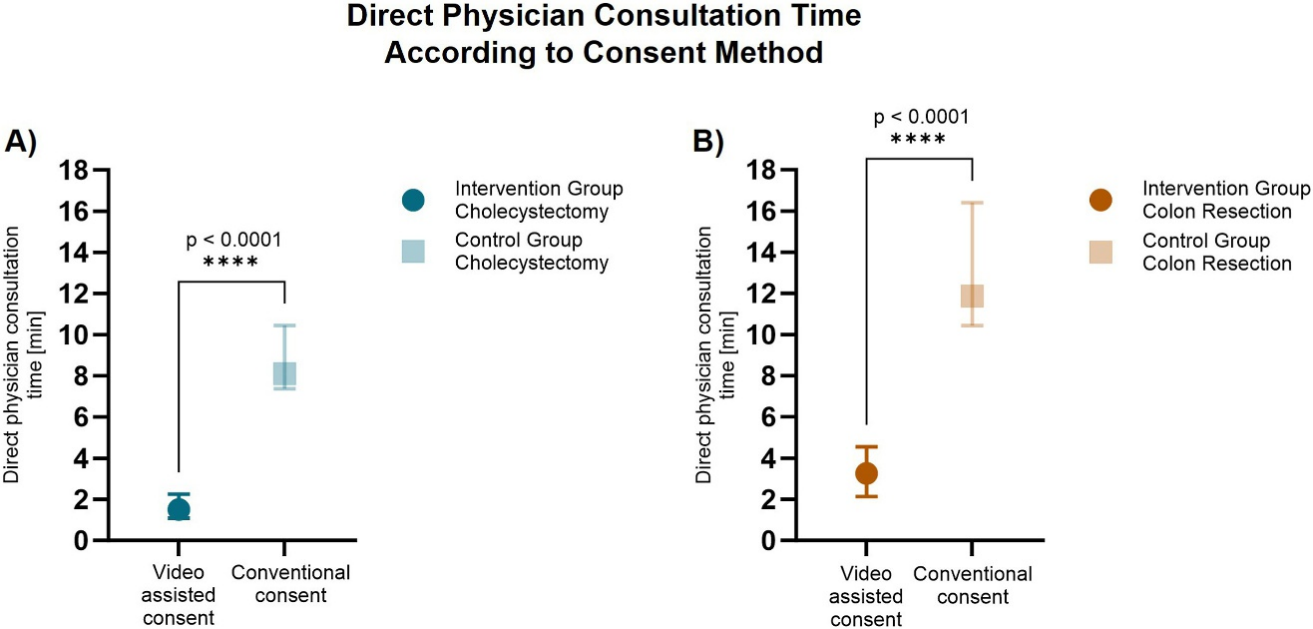
Significant time saving through the use of video‐assisted surgical informed consent. (A) The recorded physician‐led informed consent time for elective cholecystectomy after video‐assisted surgical informed consent was significantly shorter at 1.5 min [1.1–2.3] compared to the conventional informed consent time of 8.1 min [7.4–10.5] (****p < 0.0001). (B) The recorded physician‐led informed consent time for elective colon resection after video‐assisted surgical informed consent was significantly shorter at 3.3 min [2.1–4.5] compared to the conventional informed consent time of 11.9 min [10.4–16.4] (****p < 0.0001).

For patients planned for a colon resection, the median duration of the supplementary physician consultation following video presentation was 3.3 min [2.1–4.6], compared to 11.9 min [10.4–16.4] for conventional physician‐led informed consent. This corresponded to a difference of 8.6 min between the group medians (****p < 0.0001; Figure 1B).

### 3.3 High Patient‐Reported Understanding and Satisfaction in Both Groups

Patient‐reported understanding was high both preoperatively and postoperatively in the intervention and control groups for both surgical procedures (Figure 2). The statement *“I know exactly why the surgery is necessary.”* was rated as completely applicable in all subgroups before and after cholecystectomy or colon resection, irrespective of the informed consent method (Figure 2A).

**FIGURE 2:**
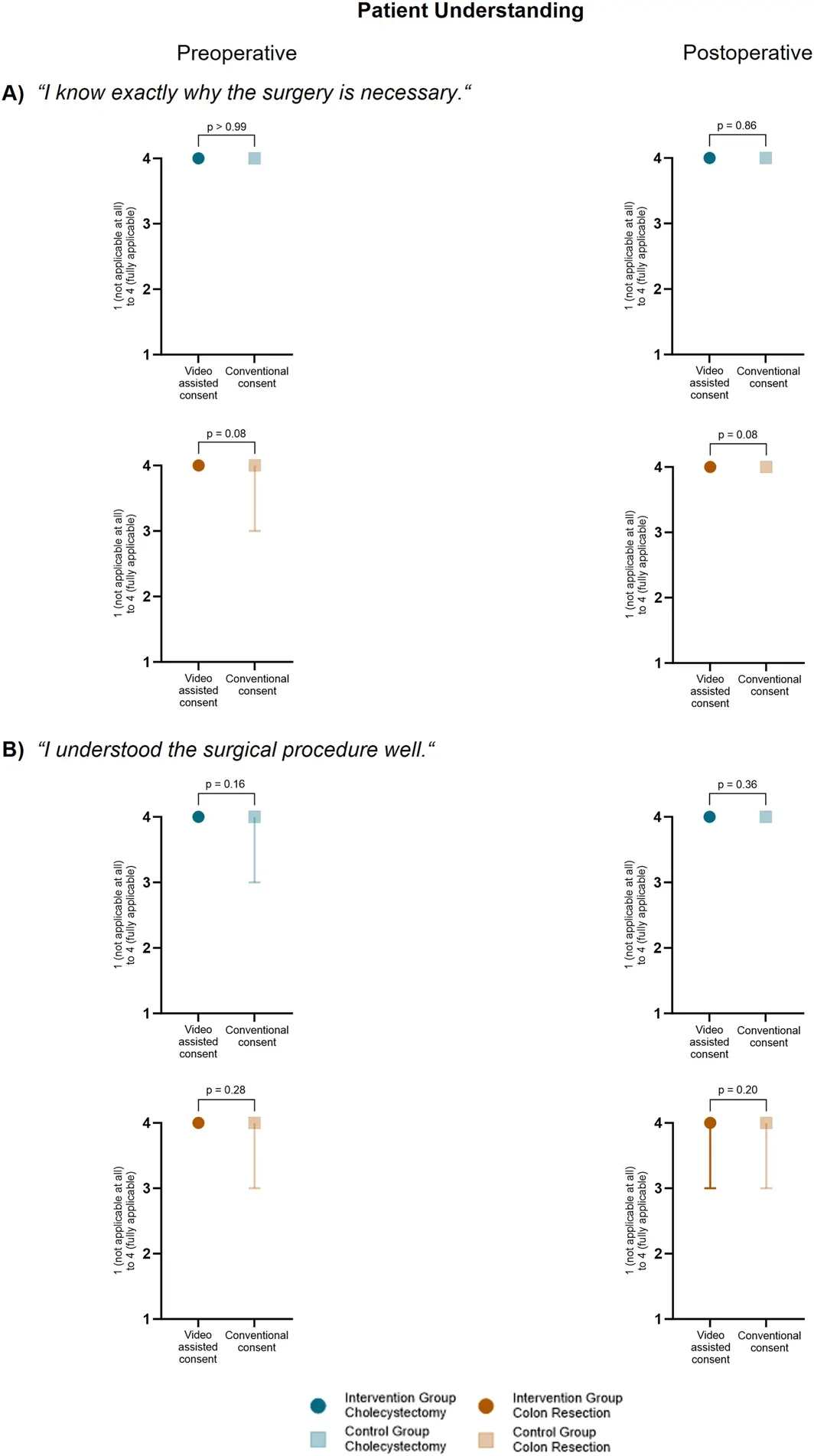
Good patient understanding regardless of surgical procedure or informed consent method. Higher scores indicate greater understanding. (A) Statement rating: *“I know exactly why the surgery is necessary.”* Prior to and following cholecystectomy, the statement was rated as nearly completely applicable by both the intervention and control groups (preoperatively: 4.0 [4.0–4.0] vs. 4.0 [4.0–4.0], *p* > 0.99; postoperatively: 4.0 [4.0–4.0] vs. 4.0 [4.0–4.0], *p* = 0.86). Prior to and following colon resection, the statement was rated as nearly completely applicable by both the intervention and control groups (preoperatively: 4.0 [4.0–4.0] vs. 4.0 [3.0–4.0], *p* = 0.08; postoperatively: 4.0 [4.0–4.0] vs. 4.0 [4.0–4.0], *p* = 0.08). (B) Statement Rating: *“I understood the surgical procedure well.”* This statement was rated as nearly completely applicable by patients in all four distinct study arms, both preoperatively and postoperatively (prior to cholecystectomy: intervention group 4.0 [4.0–4.0] vs. control group 4.0 [3.0–4.0], *p* = 0.16; following cholecystectomy: intervention group 4.0 [4.0–4.0] vs. control group 4.0 [4.0–4.0], *p* = 0.36; prior to colon resection: intervention group 4.0 [4.0–4.0] vs. control group 4.0 [3.0–4.0], *p* = 0.28; following colon resection: intervention group 4.0 [3.0–4.0] vs. control group 4.0 [3.0–4.0], *p* = 0.20).

Similarly, the statement *“I understood the surgical procedure well.”* was rated as completely applicable by patients in both the intervention and control groups before and after surgery. No statistically significant between‐group differences were detected for either cholecystectomy or colon resection (Figure 2B).

Postoperative patient satisfaction was also high in both study groups following cholecystectomy and colon resection. The item *“How would you rate the quality of the treatment you received?”* was rated as nearly excellent in all subgroups. Following cholecystectomy, the median rating was 1.0 [1.0–2.0] in the intervention group and 1.5 [1.0–2.0] in the control group (*p* = 0.45). Following colon resection, the corresponding ratings were 1.0 [1.0–2.0] and 2.0 [1.0–2.0], respectively (*p* = 0.49; Figure 3A).

**FIGURE 3:**
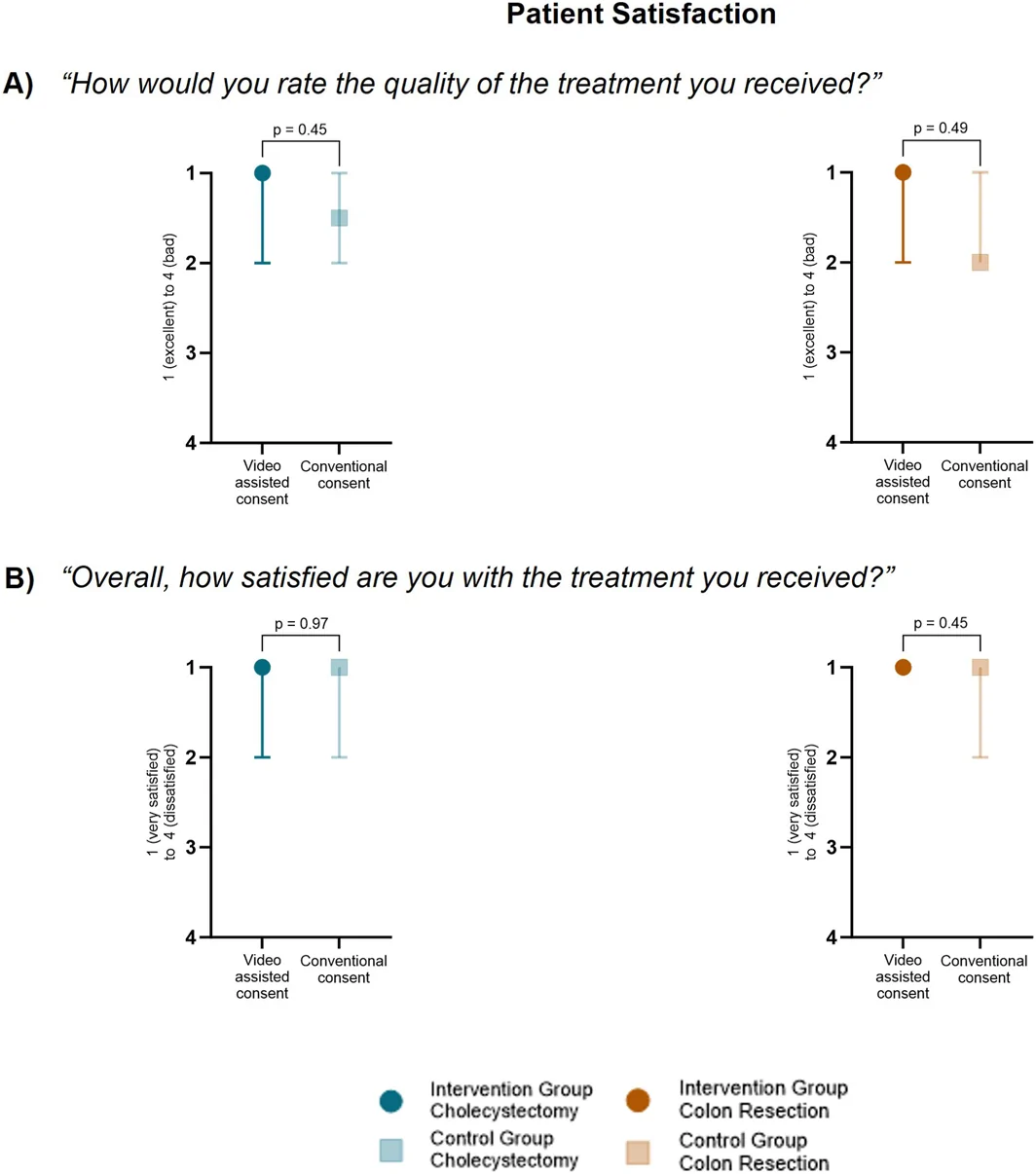
High patient satisfaction regardless of surgical procedure or informed consent method. Lower scores indicate greater satisfaction. (A) Question: “*How would you rate the quality of the treatment you received?”* Following cholecystectomy, this question was answered with a rating close to excellent in both the intervention and control groups (1.0 [1.0–2.0] vs. 1.5 [1.0–2.0], *p* = 0.45). Similarly, following colon resection, the responses were comparable between groups (intervention group: 1.0 [1.0–2.0] vs. control group: 2.0 [1.0–2.0], *p* = 0.49). (B) Question: “*Overall, how satisfied are you with the treatment you received?”* Following cholecystectomy, patients reported being nearly very satisfied in both the intervention and control groups (1.0 [1.0–2.0] vs. 1.0 [1.0–2.0], *p* = 0.97). Comparable responses were observed following colon resection (intervention group: 1.0 [1.0–1.0] vs. control group: 1.0 [1.0–2.0], *p* = 0.45).

Overall patient satisfaction, assessed using the item *“Overall, how satisfied are you with the treatment you received?”*, was similarly high across all four subgroups. Following cholecystectomy, the median rating was 1.0 [1.0–2.0] in both the intervention and control groups (*p* = 0.97). Following colon resection, the median rating was 1.0 [1.0–1.0] in the intervention group and 1.0 [1.0–2.0] in the control group (*p* = 0.45; Figure 3B).

No statistically significant between‐group differences were detected for the remaining preoperative and postoperative questionnaire items.

### 3.4 Low Postoperative Pain Scores in Both Groups

Postoperative pain was assessed using the Numeric Rating Scale (NRS) during hospitalization. Pain scores were low in both study groups, with no statistically significant between‐group differences detected for either surgical procedure.

Following cholecystectomy, the median NRS score was 1.0 [0.0–1.0] in the intervention group and 0.0 [0.0–1.0] in the control group (*p* = 0.38; Figure 4). Following colon resection, the median NRS score was 1.0 [0.0–1.0] in both groups (*p* = 0.80; Figure 4).

**FIGURE 4:**
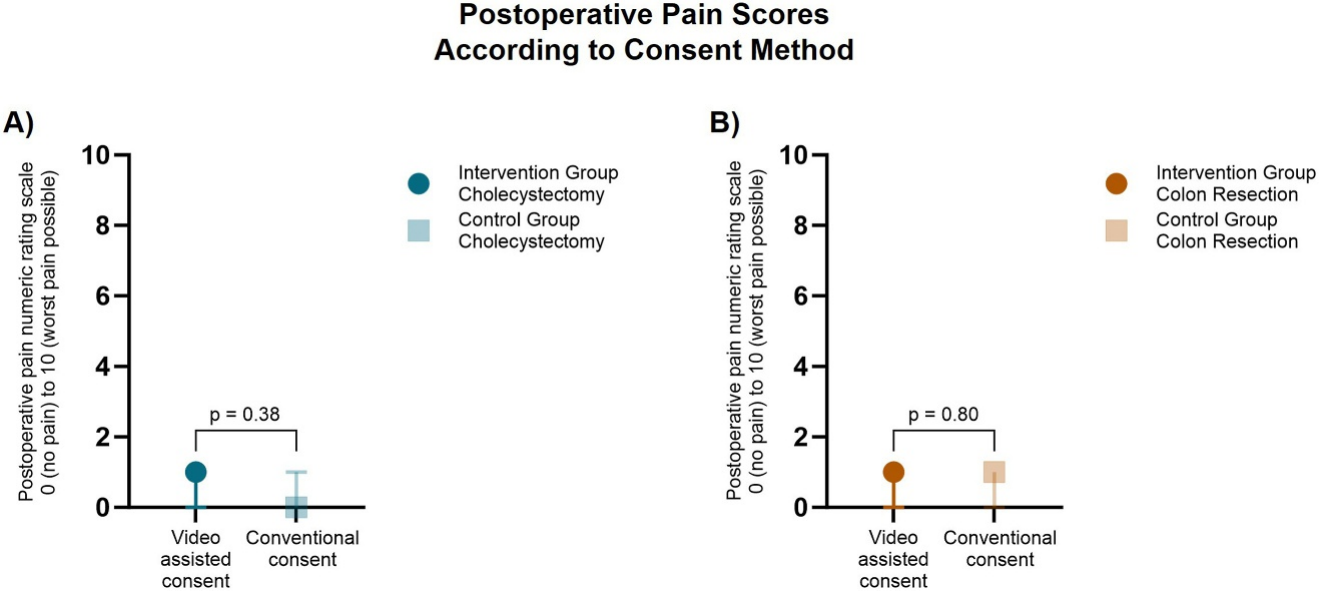
Low postoperative pain levels regardless of surgical procedure or informed consent method. (A) Postoperative pain scores did not differ significantly between the intervention and control groups following cholecystectomy (1.0 [0.0–1.0] vs. 0.0 [0.0–1.0], *p* = 0.38). (B) Postoperative pain scores did not differ significantly between the intervention and control groups following colon resection (1.0 [0.0–1.0] vs. 1.0 [0.0–1.0], *p* = 0.80).

### 3.5 Physician‐Reported Satisfaction With Video‐Assisted Informed Consent

After completion of the study, physicians who had conducted both video‐assisted and conventional informed consent procedures completed a questionnaire assessing their satisfaction with the video‐assisted approach, the number of video‐assisted consent procedures performed, and the time required to generate each individualized video.

The statement *“Overall, I was satisfied with the video‐assisted surgical informed consent.”* was predominantly rated as applicable (Figure 5A). The statement *“I found the process of conducting the video‐assisted surgical informed consent to be easy.”* was also predominantly rated as applicable (Figure 5B). The statement *“The design of the videos was appealing and an improvement over drawings and representations used in conventional informed consent.”* was predominantly rated as completely applicable (Figure 5C).

**FIGURE 5:**
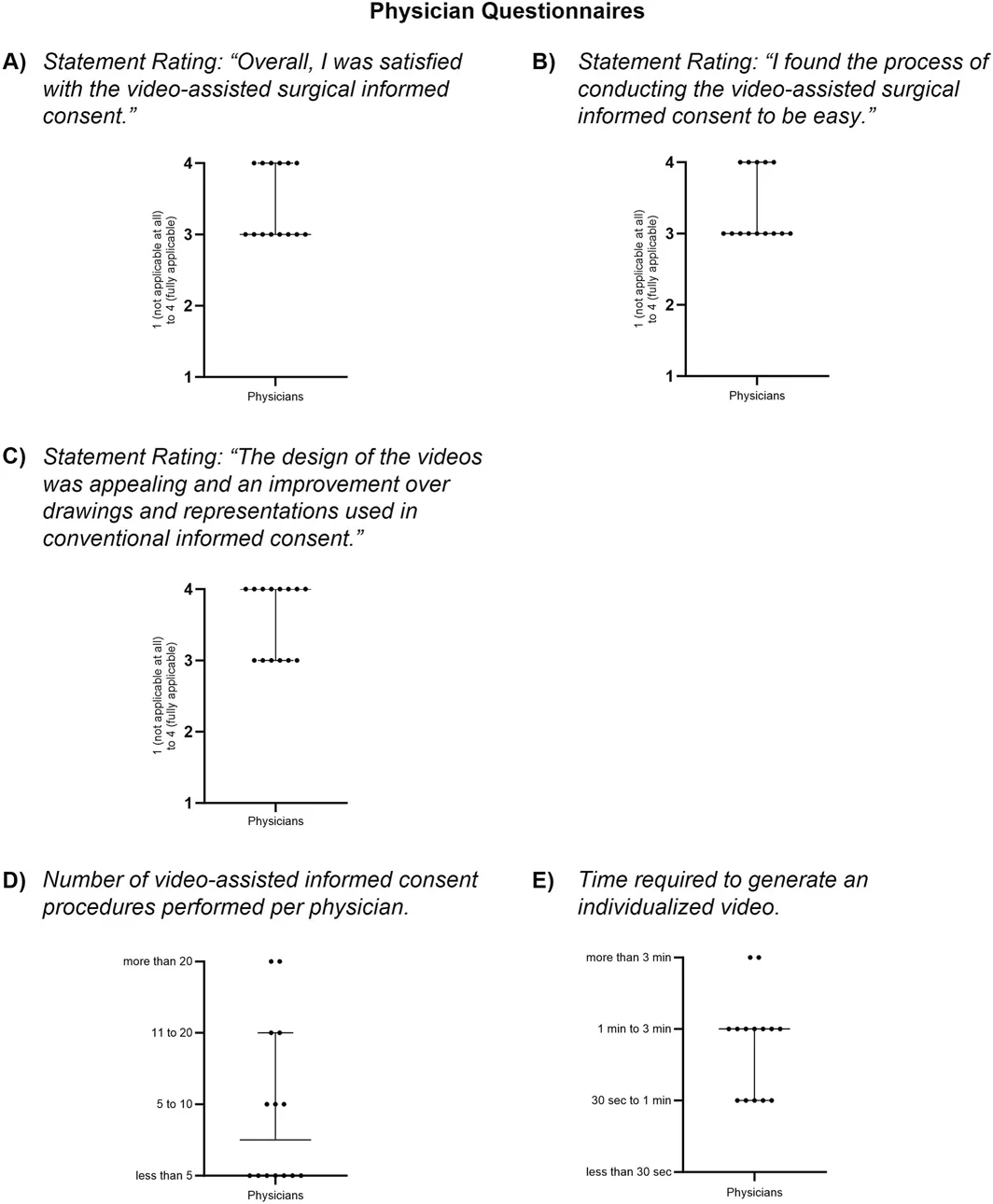
High satisfaction of physicians with video‐assisted surgical informed consent. Responses from *n* = 14 physicians. (A) Statement rating: *“Overall, I was satisfied with the video‐assisted surgical informed consent.”* The statement was rated as somewhat to completely applicable (3.0 [3.0–4.0]). (B) Statement rating: *“I found the process of conducting the video‐assisted surgical informed consent to be easy.”* The statement was rated as somewhat to completely applicable (3.0 [3.0–4.0]). (C) Statement rating: *“The design of the videos was appealing and an improvement over drawings and representations used in conventional informed consent.”* The statement was rated as nearly completely applicable (4.0 [3.0–4.0]) (D) Number of video‐assisted informed consent procedures performed per physician. Most participating physicians conducted between 5 and 10 video‐assisted informed consent procedures. (E) Time required to generate an individualized video. Most participating physicians reported requiring between 1 and 3 min to generate a single individualized video.

Most participating physicians reported having conducted between 5 and 10 video‐assisted informed consent procedures and requiring between 1 and 3 min to generate an individualized consent video (Figure 5D).

When the retrospectively reported time required to generate the individualized videos was additionally considered, the estimated physician time remained lower for video‐assisted informed consent than for conventional physician‐led informed consent for both surgical procedures (Figure 6).

**FIGURE 6:**
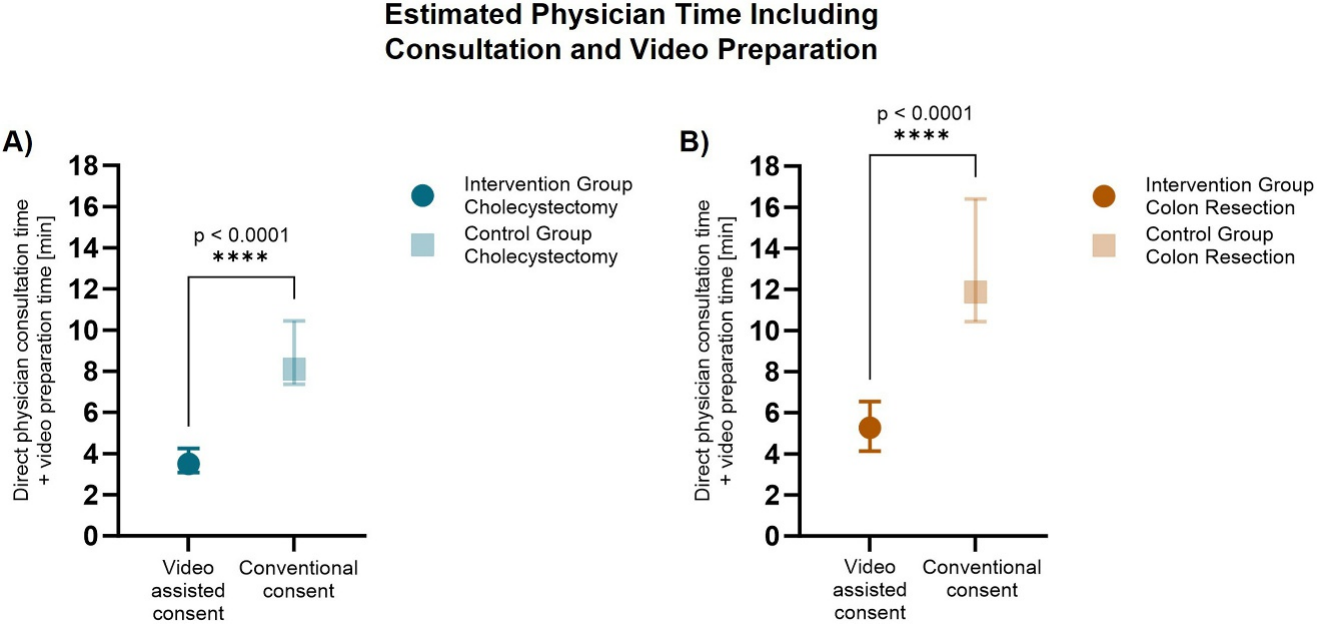
Significant time saving through the use of video‐assisted surgical informed consent after adding video‐creating time. Direct consultation time was measured prospectively, whereas video preparation time was obtained retrospectively from physician questionnaires. For the video‐assisted informed consent group, an estimated video generation time of 2 min was added to the measured physician consultation duration, corresponding to the midpoint of the 1–3 min preparation time most frequently reported by participating physicians. (A) The recorded physician‐led informed consent time for elective cholecystectomy after video‐assisted surgical informed consent with adding the video‐creating time was significantly shorter at 3.5 min [3.1–4.3] compared to the conventional informed consent time of 8.1 min [7.4–10.5] (****p < 0.0001). (B) The recorded physician‐led informed consent time for elective colon resection after video‐assisted surgical informed consent with adding the video‐creating time was significantly shorter at 5.3 min [4.1–6.5] compared to the conventional informed consent time of 11.9 min [10.4–16.4] (****p < 0.0001).

## 4 Discussion

This prospective, cluster quasi‐randomized, single‐center clinical trial showed that video‐assisted surgical informed consent was associated with a substantial reduction in direct physician consultation time. Compared to the conventional physician‐led informed consent, the between‐group difference in median consultation time was 6.6 min before elective cholecystectomy and 8.6 min before colon resection. Patient‐reported understanding and satisfaction were high in both groups, and no statistically significant between‐group differences were detected for these secondary outcomes. Postoperative pain scores were similarly low in both groups, while participating physicians reported high satisfaction with the video‐assisted consent process.

The reduction in direct physician consultation time represents the main practical implication of this study. Video‐assisted informed consent may reduce the physician time required for preoperative consent discussions and could, therefore, support more efficient clinical workflows, particularly in settings with increasing surgical case volumes and limited physician resources. Previous studies from several clinical specialties have reported potential benefits of multimedia‐assisted informed consent regarding patient understanding, satisfaction, and time efficiency [3, 12, 13, 14, 15, 16, 17, 18, 19, 20, 21, 22, 23, 24]. The present findings extend this evidence to elective visceral surgery and demonstrate a pronounced reduction in direct physician consultation time for both cholecystectomy and colon resection as representative abdominal surgery procedures, suggesting that the potential time‐saving effect may apply to procedures of different complexity [9, 10, 26, 27, 28]. The reduction in direct physician consultation time is consistent with the fact that a substantial proportion of the standardized information was delivered through the video rather than during the face‐to‐face consultation [1]. However, no formal interaction analysis was performed to determine whether the magnitude of the effect differed between the two informed consent methods. Furthermore, direct comparisons with previous studies should be made cautiously because of differences in clinical setting, video content, study design, outcome definitions, and measurement methods.

The primary outcome reflected the duration of direct physician involvement rather than the total duration of the informed consent process. The time patients spent watching the video was not measured individually, as patients could access the video at home and replay it as often as they wished. Although video generation requires additional physician time, the overall time‐saving potential of the video‐assisted approach appears to be maintained. However, as preparation time was assessed retrospectively, this finding should be interpreted with caution.

Questionnaire responses also suggested that video preparation time decreased with increasing experience. However, these findings were based on retrospective self‐report and should, therefore, be interpreted cautiously.

Patient‐reported understanding was consistently high before and after surgery in both study groups. Similarly, patient satisfaction was also high following both cholecystectomy and colon resection, with no statistically significant differences detected between the consent methods. However, understanding and satisfaction were assessed using brief self‐reported questionnaire items, and the high ratings observed in both groups may indicate a ceiling effect. The limited number of response categories may therefore have reduced the ability of the instruments to discriminate small but potentially relevant differences between the groups.

Postoperative pain may, to a limited extent, reflect patient stress and preparedness for the expected postoperative course, as adequate informed consent should help patients anticipate and understand postoperative discomfort [29]. In the present study, postoperative pain scores were consistently low after both cholecystectomy and colon resection, with no statistically significant differences between the video‐assisted and conventional informed consent groups. However, postoperative pain is influenced by numerous perioperative factors, including the type and extent of surgery, operative technique, analgesic treatment, complications, and individual pain perception.

Physician satisfaction with video‐assisted informed consent has received limited attention in previous clinical studies, although the acceptability of the intervention among healthcare professionals is relevant to its implementation. Participating physicians reported high satisfaction with the video‐assisted consent process and generally considered its implementation straightforward. These findings support the feasibility and acceptability of the intervention from the physician perspective.

Several limitations should be considered. First, allocation was performed according to predictable alternating study periods rather than by a randomly generated sequence. Allocation was not concealed, and investigators involved in patient recruitment were aware of the allocation schedule. Selection bias and temporal confounding therefore cannot be excluded. Second, patients, physicians, outcome assessors, and investigators conducting the statistical analyses were not blinded. Third, the sample size calculation was performed at the individual‐patient level and did not account for clustering within study periods. Similarly, the statistical analyses did not adjust for potential within‐period correlation. Consequently, the precision of the effect estimates may have been overestimated. Fourth, this was a single‐center study involving only elective cholecystectomy and colon resection, which limits generalizability to other hospitals, emergency procedures, and different surgical specialties. Fifth, patient understanding and satisfaction were assessed using brief self‐reported questionnaire items. Patients' baseline knowledge was not assessed before the informed consent process. Consequently, pre‐existing differences in knowledge between the groups cannot be excluded as a potential confounder. The limited number of response categories and the generally high ratings may have resulted in ceiling effects and limited the sensitivity of the instruments to detect small between‐group differences. Moreover, the study was not designed to demonstrate equivalence or non‐inferiority for these outcomes. Patients, who developed severe postoperative complications classified as Clavien–Dindo grade IV or V, were not asked to complete the postoperative questionnaires in order to avoid imposing an additional burden during critical illness. Although these patients remained included in all analyses for which data were available, their absence from the postoperative questionnaire analyses may have introduced attrition bias and contributed to an overestimation of postoperative satisfaction or other patient‐reported outcomes. Finally, patient video‐viewing time was not measured individually, while the time required to generate the individualized videos was assessed retrospectively and was not included in the prespecified primary outcome.

Nevertheless, video‐assisted informed consent offers advantages, such as the option to provide information in multiple languages, which is becoming increasingly important in light of a more international patient population [30]. The availability of these videos in home settings also addresses challenges arising from the ongoing centralization of hospital services, which often increases the geographic distance between patients and healthcare facilities [31].

Despite these limitations, the magnitude and consistency of the observed reduction in direct physician consultation time across both surgical cohorts suggest that video‐assisted informed consent warrants further evaluation. Future multicenter randomized studies should use concealed allocation, account for clustering in both sample size calculations and statistical analyses, employ validated and sufficiently sensitive measures of patient understanding and satisfaction, and prospectively assess the entire consent workflow. Economic evaluations should additionally determine whether reductions in direct physician time translate into meaningful improvements in staffing efficiency or healthcare costs.

## 5 Conclusion

In conclusion, video‐assisted surgical informed consent was associated with a substantial reduction in direct physician consultation time compared to the conventional physician‐led informed consent. Patient‐reported understanding and satisfaction were high in both groups, with no statistically significant between‐group differences detected; however, the study was not designed to establish equivalence or non‐inferiority for these outcomes. Participating physicians also reported high satisfaction with the video‐assisted approach. These findings support the feasibility of video‐assisted informed consent as a complementary approach to preoperative patient education and are consistent with the broader digital transformation of healthcare. Further multicenter randomized studies are required to assess the entire consent workflow, patient‐reported outcomes, and economic implications.

## Author Contributions

**Svenja G. P. D. Leicht:** writing – original draft, investigation, conceptualization, methodology, validation, visualization, writing – review and editing, software, formal analysis, project administration, data curation. **Leon Schuetze:** conceptualization, investigation, software, project administration, methodology, validation, writing – review and editing. **Johan F. Lock:** conceptualization, investigation, funding acquisition, methodology, validation, writing – review and editing, supervision, data curation, resources, formal analysis. **Christoph–Thomas Germer:** funding acquisition, conceptualization. **Johanna C. Wagner:** conceptualization, investigation, writing – review and editing, methodology, data curation, supervision, resources, project administration, formal analysis, software, validation.

## Funding

*Medudoc education GmbH* made the videos available free of charge during the period of the clinical trial. This publication was funded by the University of Wuerzburg in the funding program Open Access Publishing.

## Ethics Statement

These study results were presented at the Visceral Medicine Congress in Leipzig, Germany, in October 2024 and at the Bavarian Surgeons Congress in Regensburg, Germany, in July 2025.

## Conflicts of Interest

The videos for performing the video‐assisted surgical informed consent were provided by *medudoc education GmbH*.

## Data Availability

The data that support the findings of this study are available from the corresponding author upon reasonable request.
